# Analysis of Calcium Patterns in the Thoracic Aorta and Clinical Outcomes of TAVR Patients Presenting with Porcelain Aorta

**DOI:** 10.3390/jcm14020503

**Published:** 2025-01-14

**Authors:** Caterina Campanella, Stephanie Voss, Julia Schreyer, Nazan Puluca, Andrea Amabile, Felix Wirth, Markus Krane, Hendrik Ruge

**Affiliations:** 1 Department of Cardiovascular Surgery, Institute Insure, German Heart Center Munich, School of Medicine & Health, Technical University of Munich, Lazarettstrasse 36, 80636 Munich, Germany; campanella@dhm.mhn.de (C.C.); vosss@dhm.mhn.de (S.V.); schreyer@dhm.mhn.de (J.S.); puluca@dhm.mhn.de (N.P.); amabile.andrea@gmail.com (A.A.); wirth@dhm.mhn.de (F.W.); 2 Division of Cardiac Surgery, Department of Cardiothoracic Surgery, University of Pittsburgh, Pittsburgh, PA 15260, USA; 3 UPMC Heart and Vascular Institute, University of Pittsburgh Medical Center, Pittsburgh, PA 15219, USA; 4 DZHK (German Center for Cardiovascular Research), Partner Site Munich Heart Alliance, 81377 Munich, Germany; 5 Division of Cardiac Sugery, Department of Surgery, Yale School of Medicine, New Haven, CT 06510, USA

**Keywords:** TAVR, aortic arch, porcelain aorta, calcifications

## Abstract

**Background/Objectives**: In the presence of porcelain aorta (PA), transcatheter aortic valve replacement (TAVR) has become a class I therapeutic indication for the treatment of severe aortic valve stenosis. To date, few studies have analyzed the clinical outcomes of TAVR in PA patients. We aim to analyze the calcification patterns of the thoracic aorta in PA patients and to evaluate their clinical implications for TAVR procedures. **Methods**: This study included 161 patients who had PA confirmed through pre-operative CT and underwent TAVR between 11/2014 and 12/2022. The primary outcome was to perform a multi-slice CT (MSCT) analysis assessing the calcification in the proximal, middle, and distal thoracic aortic segments. Each segment was divided into quadrants for scoring calcifications on a scale from 1 (<25%) to 4 (>75%). The cohort was categorized into circular or noncircular calcification group. The secondary clinical outcomes were defined according to VARC-3 criteria. **Results**: The study cohort included 161 patients (median age, 77.2 years; IQR, 70.1–82.6 years). The median EuroSCORE II and STS predicted risk of mortality were 3.10% [1.80–5.58] and 2.70% [1.70–4.30], respectively. In 75% of patients (n = 121/161), TAVR was performed via transfemoral access. Circular calcifications were found in 8.1% of patients, while noncircular calcifications were present in 91.9%. Significant calcifications were primarily in the right quadrant of the proximal segment (33.54%), superior quadrant of the middle segment (39.75%), and left quadrant of the distal segment (73.29%). The 30-day mortality rate was 3.11% and periprocedural ischemic stroke rate 3.38%. **Conclusions**: Most TAVR patients with PA exhibited noncircular calcification. The most extensive calcifications were primarily in areas relevant to surgical manipulation. Patients with PA displayed low short-term mortality and relatively few stroke events. In view of these findings, TAVR constitutes a valid treatment option for patients with PA and aortic stenosis.

## 1. Introduction

Transcatheter aortic valve replacement (TAVR) is a safe and reproducible technique for treating severe aortic valve stenosis in high-, moderate-, and low-risk patients [1,2,3,4,5]. According to the 2021 guidelines of the European Society of Cardiology/European Association for Cardio-Thoracic Surgery (ESC/EACTS), TAVR is the preferred treatment for patients with extensive aortic calcifications, also known as porcelain aorta (PA) [6]. PA is defined as a structural aortic wall disease characterized by substantial calcific build-up within the ascending thoracic aorta, extending into the aortic arch as well as the descending aorta [1,2,5]. Its reported incidence varies across the literature, but it can be as high as 7.5% in patients requiring cardiac surgery [1,7], while reaching 18% in patients with aortic stenosis (AS) [4]. Excessive aortic calcification poses a considerable challenge to aortic cross-clamping or arterial cannulation during cardiac surgery [1,2], increasing the risk of perioperative embolic stroke and aortic dissection [1,2,4,5]. Therefore, PA is considered a relative contraindication for surgical aortic valve replacement (SAVR) [5]. Meanwhile, transcatheter aortic valve replacement (TAVR) is recommended as a viable and safe alternative option [1,2,3,4,5].

Previous studies on PA have cited its presence as the sole indication for TAVR, regardless of SAVR-related operative risk. In fact, among patients < 70 years of age who present with PA and AS, TAVR has prevailed over SAVR [8]. As PA increasingly becomes a pivotal independent factor in patient selection, and, particularly with the expansion of transcatheter aortic valve replacement (TAVR) to younger and lower-risk surgical groups, it is essential to enhance our understanding of PA and its clinical implications in TAVR procedures.

However, data on thoracic aortic calcium distribution/morphology and on clinical outcomes of TAVR procedures in PA patients are sparse. Therefore, we conducted the present computed tomography (CT)-based analysis of thoracic aortic calcium localization pattern and analyzed the clinical outcomes of TAVR patients presenting with porcelain aorta.

## 2. Materials and Methods

### 2.1. Patient Population

**Figure 1:**
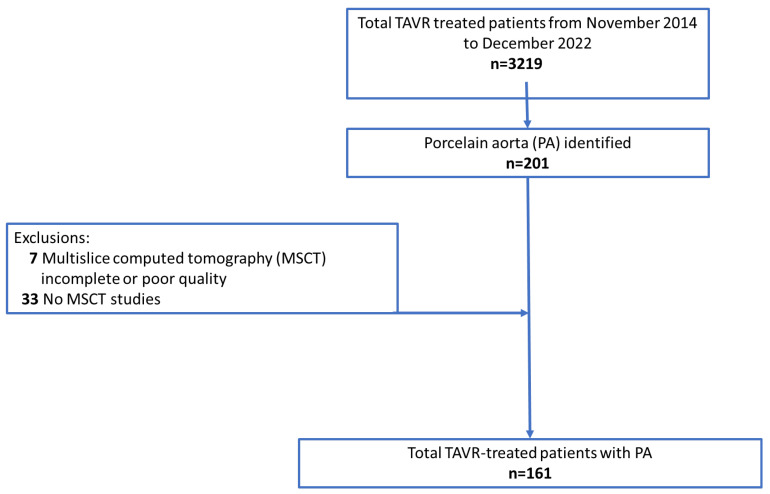
Flowchart of patient selection after transcatheter aortic valve replacement (TAVR) procedures.

Our cohort consisted of all consecutive patients with severe, symptomatic, native AS with a history of PA and who underwent TAVR at the Department of Cardiovascular Surgery at the German Heart Centre Munich between November 2014 and December 2022. Inclusion criteria were available pre-TAVR computed tomography (CT) studies and use of the latest generations of transcatheter heart valves (THVs), including Portico (Abbott Laboratories, Abbott Park, IL, USA), Sapien 3 or Sapien 3 Ultra (Edwards Lifesciences, Irvine, CA, USA), Evolut R or Evolut PRO (Metdronic, Minneapolis, MN, USA), and Acurate neo 2 or Lotus (Boston Scientific, Watertown, MA, USA) devices. Unavailable, incomplete, or poor quality multi-slice CT (MSCT) datasets of the aortic arch and its supra-aortic branches (Figure 1) were reasons for study exclusion.

Baseline characteristics such as risk scores, comorbidities, MSCT measurements, and echocardiographic data were prospectively recorded in a dedicated database, as were procedural data and perioperative complications. Follow-up data were collected from recent medical reports, acquired at outpatient visits, or obtained by telephone interviews.

The study complied with the Declaration of Helsinki and was approved by the local ethics committee of the Technical University of Munich (approval reference number: 2024-87-S-CB).

### 2.2. Outcomes Analysis

The primary outcome was to perform a CT-based analysis to evaluate the calcium distribution and patterns in the thoracic aorta of patients undergoing the TAVR procedure. Secondary outcomes were 30-day all-cause mortality, 30-day stroke, device success at 30 days, and the early safety combined endpoint at 30 days, according to the recent VARC-3 document.

### 2.3. MSCT Measurements of Ascending Aorta and Aortic Arch

We performed a retrospective analysis of all pre-TAVR MSCT findings using 3mensio Structural Heart (v10.2; Pie Medical Imaging, Maastricht, the Netherlands) software for automated 3-dimensional CT reconstruction. MSCT studies served to confirm the presence of PA and define calcium distributions, as previously described [9]. After selecting the aortic arch on the subclavian route assessment module, a central line coursing along its path is automatically generated. This linear depiction allows up-and-down scrolling to assess calcium patterns in pre-specified segments. The protocol we followed for calcium pattern determination and semiquantitative scoring has been previously detailed by Snow, et al. [9].

The protocol we followed for calcium pattern determination and semiquantitative scoring was based on the previously detailed protocol by Snow et al. [9].

The study by Snow et al. defined the highest point on the scale as 100% calcification involvement. We decided to limit the scale to point number 4 (>75–100%), as the clinical implications of calcium involvement do not differ above 75%.

### 2.4. Ascending Aorta and Aortic Arch Calcium Patterns

**Figure 2:**
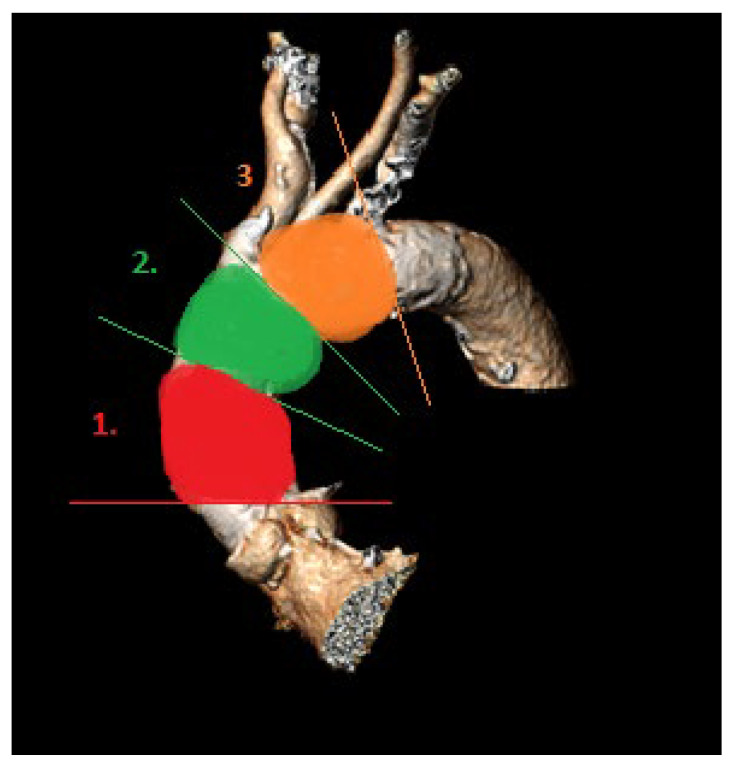
Aortic arch segments (1, proximal; 2, middle; 3, distal) for calcium pattern analysis.

For calcium pattern analysis, the ascending aorta and the aortic arch were divided into three contiguous segments (Figure 2) as follows:

(1) Proximal segment, from sinotubular junction to mid-ascending aorta (at level of pulmonary artery bifurcation);
(2) Middle segment, from mid-ascending aorta (at pulmonary artery bifurcation) to origin of brachiocephalic trunk;
(3) Distal segment, from origin of brachiocephalic trunk to origin of left subclavian artery.

**Figure 3:**
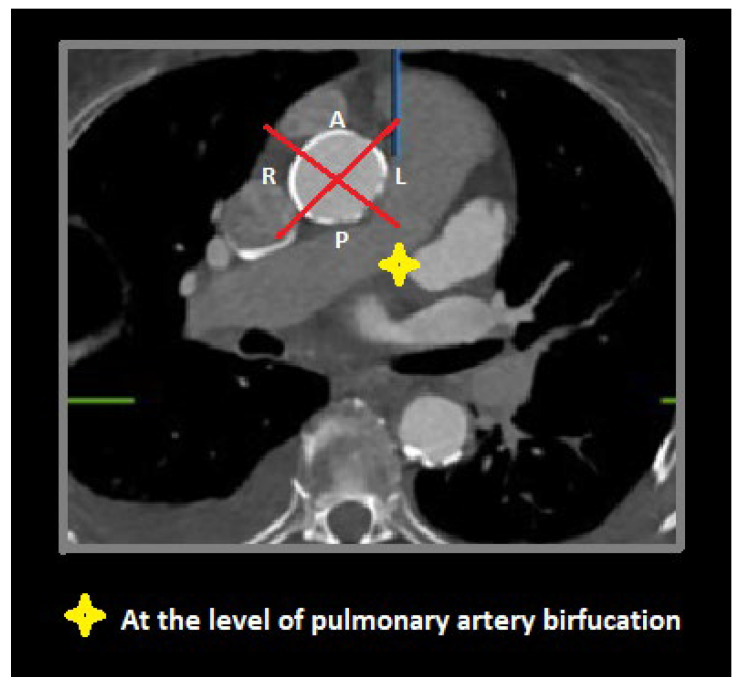
Axial view through proximal aortic segment (at pulmonary artery bifurcation) demonstrating anterior (A), left (L), posterior (P), and right (R) quadrants.

**Figure 4:**
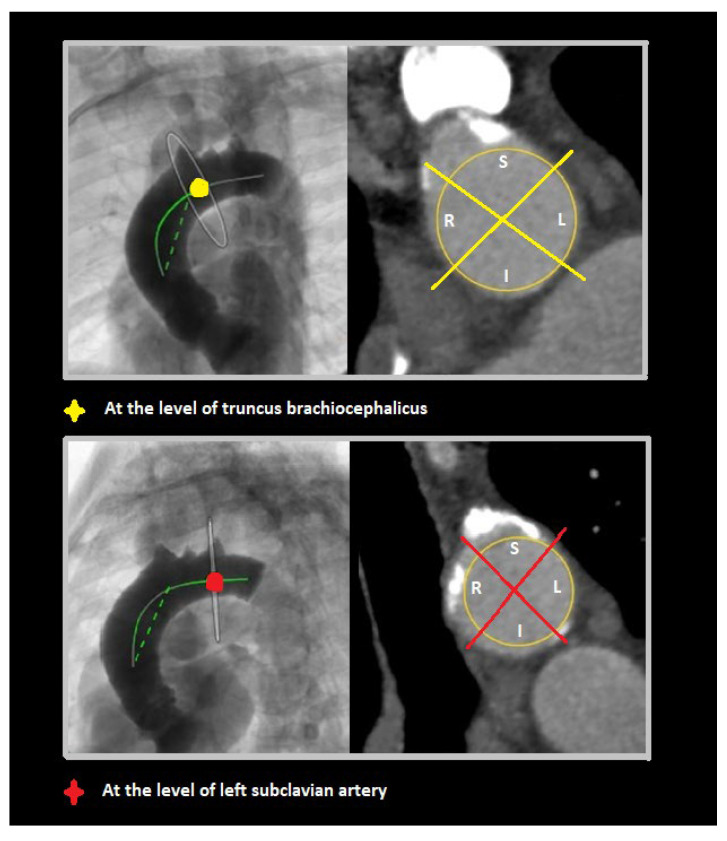
Perpendicular views of middle (at truncus brachiocephalicus) and distal (at left subclavian artery) aortic segments, with superior (S), left (L), inferior (I), and right (R) quadrants shown in each.

To visually assess calcium distributions, each aortic segment was further partitioned on perpendicular view into quadrants referencing bodily orientation. For the proximal segment, these quadrants were designated anterior, left, posterior, and right (Figure 3). In the middle and the distal segments, defined quadrants were superior, left, inferior, and right (Figure 4).

Based on visual approximations of proportionate calcific involvement, we scored each quadrant of the three defined aortic segments as follows: 0, no calcifications; 1, <25% of aortic wall showed calcification; 2, 25–49% of aortic wall showed calcification; 3, 50–74% of aortic wall showed calcification; and 4, 75–100% of aortic wall showed calcification. The mean score for each segment was calculated as the sum of its quadrant scores, divided by 4.

### 2.5. Group Assignments

**Figure 5:**
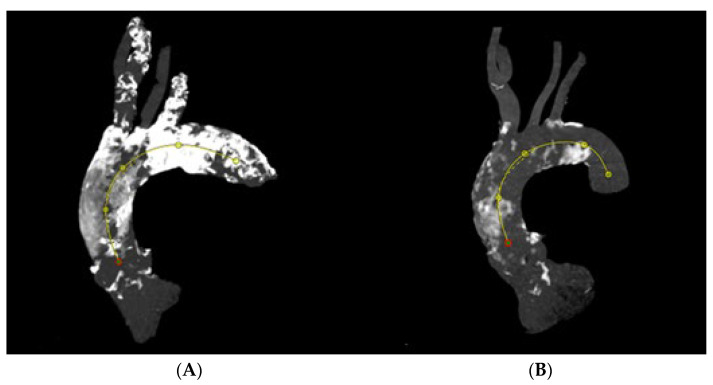
Circular calcification along thoracic aorta (**A**), noncircular calcification of thoracic aorta (**B**).

We assigned study subjects to one of two groups, reflecting the nature of aortic calcific involvement. The circular group included patients presenting with >50% calcification of every quadrant in each aortic segment. In the noncircular group, not every quadrant of the various segments was involved to such extent (Figure 5).

### 2.6. Clinical Data Analysis

Demographic, laboratory, procedural, and postoperative data were retrieved from our institutional TAVR database, which adhered to the latest Valve Academic Research Consortium 3 (VARC-3) standards. Baseline patient characteristics included age, sex, body mass index (BMI), European System for Cardiac Operative Risk Evaluation II (EuroScore II) status, log EuroScore, and Society of Thoracic Surgeons (STS) predicted mortality risk. We also examined various comorbidities, specifically coronary artery disease, prior percutaneous coronary intervention (PCI) or coronary artery bypass graft (CABG), peripheral arterial disease (PAD), carotid artery disease (CAD), chronic kidney disease, chronic obstructive lung disease (COPD), history of stroke, pacemaker implantation, atrial fibrillation (AF), and past radiation therapy. Preoperative echocardiographic data were collected prospectively.

Procedural variables included intervention time, embolic protection device usage, balloon pre- and post-dilatation, intraoperative vessel-related or bleeding adverse events, vascular access site, and type of THV. Technical success at discharge was defined according to VARC-3 criteria.

Strokes were defined as focal neurologic signs/symptoms of acute onset, linked to one or more vascular territories within the brain, spinal cord, or retina and associated with lingering symptoms (>24 h) or neuroimaging evidence of central nervous system (CNS) infarction. Follow-up was achieved through medical record reviews and telephone calls.

### 2.7. Statistical Analysis

Continuous variables were expressed as mean and standard deviation values or median and interquartile ranges based on normalcy of distribution, while categorical variables were expressed as absolute numbers with percentages. The Shapiro–Wilk test was implemented to test continuous variables for normal distribution, using a two-sided *t*-test or Mann–Whitney *U* test accordingly in group comparisons [10]. We analyzed time-to-event outcomes by the Kaplan–Meier method. All statistical calculations were performed using the R statistical computing environment (version 4.2.1), setting significance at *p* < 0.05.

## 3. Results

### 3.1. Baseline Characteristics

Between November 2014 and December 2022, a total of 3129 patients underwent TAVR procedures in the Department of Cardiovascular Surgery at the German Heart Center Munich. Among them, 201 displayed features of PA. A total of 40 patients were excluded due to MSCT issues (incomplete or poor quality [n = 7]; no available scans [n = 33]); 13 patients (n = 13 of 161, 8.1%) presented with circular aortic calcifications (circular group). The vast majority (n = 148 of 161, 91.9%) were marked by noncircular calcific patterns (noncircular group). The median age at the time of TAVR was 77.2 years (range, 70.1–82.6 years), and the median EuroSCORE II and STS mortality risk score were 3.10% (95% CI: 1.80–5.58) and 2.70% (95% CI: 1.70–4.30), respectively. History of radiation was significantly more frequent in the circular aortic calcification group (38.5%, [n = 5 of 13] vs. 9.46% [n = 14 of 148]; *p* = 0.012). Preoperative echocardiographic data showed no difference between the two groups. Detailed results are listed in the Supplementary Table S1. Further baseline characteristics were comparable among both groups and are displayed in Table 1.

### 3.2. Calcium Distribution and Morphology

**Figure 6:**
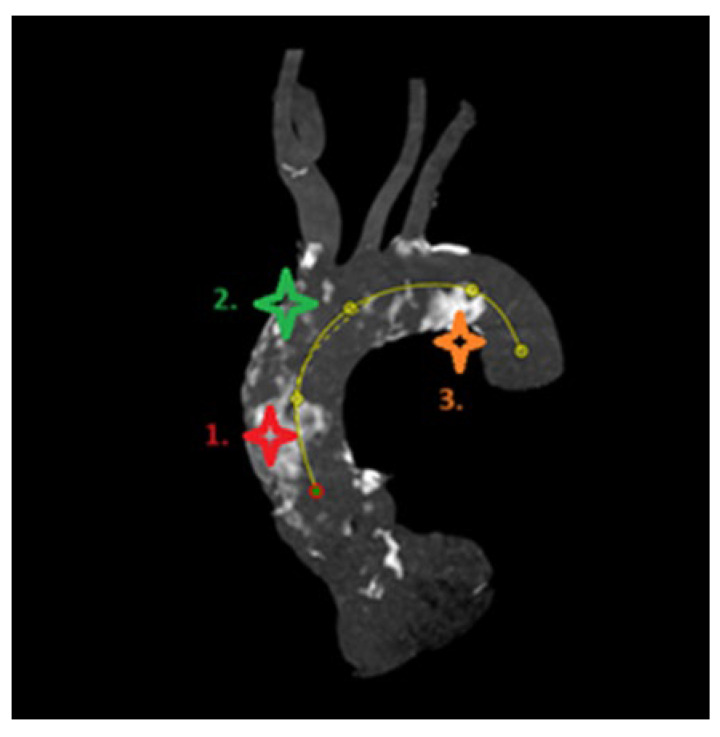
Predominant localization of calcium in previously defined aortic arch segment: nr1. right quadrant, nr2. superior quadrant, nr3. left quadrant.

Our MSCT measurements revealed that the distal aortic segments contained the highest calcium load, categorized as a calcium score grade 4 (maximum value) in 46.6% of patients (Table 2). Calcium analysis revealed maximal scoring in certain quadrants of proximal (left, 26.71%; right, 33.54%), middle (right, 32.3%; superior, 39.75%), and distal (superior, 59.01% inferior, 55.28%; left, 73.29%) segments (Figure 6). Details of calcium distribution patterns are provided in Table 3.

### 3.3. Procedural Data and Clinical Outcomes

Transfemoral access (n = 121 of 161, 75.2%) was the prevailing approach in our cohort, followed by transapical (n = 22 of 161, 13.7%), transaortic (n = 10 of 161, 6.21%), and subclavian (n = 8 of 161, 4.97%) routes. A total of 101 patients (62.7%) received balloon-expandable valve (BEV) (circular group: n = 12 of 13, 92.3%; noncircular group: n = 89 of 148, 60.1%), whereas 60 patients (circular group: n = 1 of 13, 7.69%; noncircular group: n = 59 of 148, 39.9%; *p* = 0.032) were treated with self-expanding valves (SEVs). A detailed description of the types of transcatheter heart valves (THVs) is provided in Supplementary Table S3. Overall, pre- and post-dilatation was performed in 30.4% and 34.8%, respectively, without differences between the groups. TAVR access routes, intervention times, and intraoperative complications were similar among both groups (Table 4).

No major cardiac structural injury occurred in the overall cohort. In the noncircular group, 22 patients (14.9%) had major vascular access complications, as opposed to one patient (7.69%) in the circular group (*p* = 0.764). Major bleeding events were confined to 14 patients (9.46%) of the noncircular group (*p* = 0.607). A total of 14 patients (n = 14 of 161, 8.7%) did require surgical interventions for major bleeding complications.

Periprocedural clinical details after TAVR procedures are displayed in Table 5.

**Figure 7:**
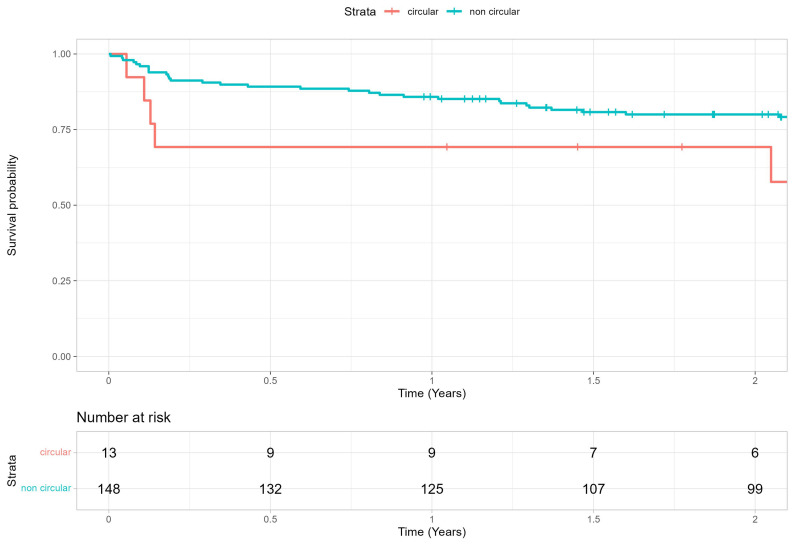
Two-year survival rate after TAVR procedure in PA patients presenting with circular calcification patterns and noncircular calcification patterns.

Overall device success at discharge was 85.7%. There were no significant group differences in 30-day mortality rates (circular group: n = 1 of 13, 7.69%; noncircular group: n = 3 of 148, 2.03%; *p* = 0.347). Survival at 2 years was 79.1 ± 3.2% overall, 80.0 ± 3.3% and 69.2 ± 12.8% for the noncircular and circular group, respectively (Table 6, Figure 7). Postoperative echocardiographic data are shown in the Supplementary Materials (Supplementary Table S2).

## 4. Discussion

The main findings of the present analysis of TAVR patients with PA (n = 161) are the following: (1) Circular calcification patterns of ascending aorta and aortic arch were infrequent (8%) in PA patients undergoing TAVR; (2) the calcium mainly accumulated (scored as 4) in the superior (59.28%) and left (73.29%) quadrants of the distal aortic segments, with the superior (39.75%) and right (32.3%) quadrants of the middle segments and the right (33.54%) and left (26.71%) quadrants of the proximal segment being less affected; (3) the 30-day postoperative stroke rate was 3.1% and did not differ significantly by calcification group; (4) mortality at 30 days was low (3.11%), and device success at discharge was 85.7%.

### 4.1. Calcium Distribution

There is no standard accepted definition for PA. Interpretations vary from one author to another [1,5], ranging from brief mention of a generally “severe atherosclerotic ascending aorta” [2] to more elaborate portrayals, stipulating “circumferential calcification or severe atheromatous plaques of the entire ascending aorta, extending to the arch such that aortic cross clamping is not feasible” [5]. However, the common denominator for PA is its clinical interference with surgical treatment. Related studies have shown that patients presenting with PA and undergoing cardiac surgery bear higher morbidity and mortality rates, due to increased risk of embolic stroke [1,2]. Such complications are usually attributable to manipulation of heavily calcified plaques in atheromatous ascending aortas during cannulation and clamping. Hence, such patients are instead diverted to interventional procedures, rather than to surgical procedures, although PA may also increase the complications risk for interventional procedures [1,11,12,13].

The impact of PA on cardiac surgery has been well established, but PA may also increase the complications risk for interventional procedures [1,12,13]. PA influence on TAVR outcomes is not well studied yet. Recent studies have investigated postprocedural clinical outcomes of TAVR patients with PA, although without focusing on the severity of PA and its influence of perioperative complication rate. Our study used of a pre-defined and reproducible protocol for pre-TAVR MSCT data evaluation, enabling the present analysis of thoracic aortic calcium patterns in patients with PA. We were therefore able to localize and quantify the extent of calcific change in diseased aortas. Heavy calcification (scored as 4) was shown to predominate along the outer curvature of aortic arch and the superior and right aspect of ascending aorta, at the origin of the brachiocephalic trunk. This anatomic zone corresponds with sites of cannulation and aortic clamping during cardiac surgery, underscoring the true surgical impediment of PA. Our findings are in agreement with those generated by Snow et al., having similarly demonstrated slight predominance of calcium distribution in the superior quadrant of transverse aorta in TAVR patients [9]. These findings support the choice of TAVR over SAVR in PA patients.

### 4.2. Stroke Rate

It is generally acknowledged that TAVR carries a higher risk of stroke (relative to the general population) for up to 2 years thereafter [14]. Use of a self-expandable THV and balloon post-dilatation during procedures are other factors that increase this risk [14,15,16]. Despite the intuitive predisposition to embolic stroke caused by the valve delivery system traveling through the diseased aorta, we did not observe a higher incidence of stroke in relation to all-comers analysis [17]. The PARTNER 2 trials have reported a 30-day all-stroke rate of 4.2% after TAVR [17]. These findings are in line with our 30-day stroke rate of 3.1% in TAVR-patients with PA. Eckel et al. compared 492 TAVR-patients with PA with 984 TAVR-patients without PA and found a similar stroke incidence after 30 days of 2.7% in PA patients after TAVR [18]. Still, there are conflicting data from earlier studies, citing higher rates of periprocedural stroke in patients with (vs. without) PA who undergo TAVR. Asami et al. have recently reported a higher incidence of disabling stroke at 1 year after TAVR in the presence (vs. absence) of PA (7.2% [n = 8] vs. 3.0% [n = 61]; *p* = 0.03) [5].

### 4.3. Mortality Rate and Clinical Outcomes

In 2013, a German TAVR registry sought to compare 147 patients (10.7%) with PA and 1227 patients (89.3%) without PA. The 30-day mortality rate (10.9% vs. 8.1%; *p* = 0.24) and in-hospital combined death/stroke rate (14.4% vs. 10.2%; *p* = 0.12) were higher in PA than in non-PA patients [19]. The 30-day mortality rate (3.11%) of our cohort was distinctly lower, perhaps given the comparatively lower mean age (77.2 vs. 81.4 ± 6.2 years) [19]. Four patients died from cardiovascular causes after discharge (three from the noncircular group and one from the circular group). Only one patient died due to non-cardiovascular causes from the noncircular group.

Furthermore, we found no significant group differences in major vascular and access complications, pacemaker implantations, or major bleeding events at discharge, knowing the substantial calcific burden of vessel perimeter (>75%) in the circular group.

Although our study demonstrated a low mortality rate, we acknowledge the importance of minimizing postoperative complications. To further reduce these complications, we could focus on optimizing specific aspects of the TAVR procedure. For instance, ultrasound-guided vascular puncture could help identify the optimal access point by selecting areas with less calcification, thereby reducing the risk of vessel injury and improving sheath insertion. Additionally the use of steerable and flexible catheters may reduce wall contact, lowering the risk of calcium dislodgement and stroke. Newer generations of THV devices, with enhanced steering capabilities and smaller diameters, could improve procedural success, even in the presence of severe calcifications, by facilitating smoother vessel navigation and more precise device deployment.

For the entire cohort, early device success at discharge was 85.09%. This result can be favorably compared with that of Eckel et al. showing similar device success at discharge with a rate of 81.5%, although only for self-expandable THV in PA patients [18]. It then appears that TAVR in the setting of PA may yield procedural and clinical outcomes comparable to those achieved in the absence of PA.

### 4.4. Method of Vascular Access

The transfemoral-first strategy has become widely adopted and is currently considered the gold standard for TAVR [20]. In 2021, the German Registry recorded a 93.1% rate of transfemoral access [21]. The analogous rates for transfemoral access for our circular (n = 10 of 13, 76.9%) and noncircular (n = 111 of 148, 75.0%) patient groups were significantly lower than the findings reported from the German Registry and may be explained by time frame disparity. The proportion of patients with transfemoral TAVR has risen exponentially between 2013 and 2019, with femoral access accounting for 95% of all TAVR procedures in 2019 [22]. Indeed, 65% of our procedures with non-transfemoral access were executed before 2019 with predecessor models of the current devices, which had larger delivery system diameters and poorer steerability, more often requiring vascular access other than transfemoral. Although an antegrade, transapical approach has been suggested in cases of PA to minimize contact with the aortic wall and avoid endothelial damage [23], there is no objective data to date demonstrating the superiority of this approach. To the contrary, alternative access routes have been linked to a three-fold increase of periprocedural disabling stroke risk after TAVR in patients with PA [5]. We used alternative access routes in 24.8% of patients with no particular correlation between access (i.e., transfemoral vs. other route) and stroke rate.

### 4.5. Future Perspective

Ultimately, the calcifications pattern within ascending aorta and aortic arch found in our cohort resulted in similar perioperative complications, compared with previous all-comers studies [17]. To better address questions on calcium burden and safety of TAVR procedures in patients with PA, further research into calcium distributions is needed. This might lead to a better definition for PA following more standardized reporting of future research on PA patients. This is a topic of clinical importance, especially in younger patients (<75 years) for whom the presence of PA is often the sole basis of prioritizing TAVR over SAVR. Another worthy area of pursuit is the use of embolic protection devices to prevent strokes in the setting of PA. Randomized trials with broader patient samplings should help to determine the actual clinical benefits derived in a cohort such as ours.

## 5. Limitations

The present study was a retrospective, single-center investigation with a relatively small sample size. Our study primarily reflects perioperative outcomes in a limited number of patients with porcelain aorta. A key limitation is the lack of a control group of patients without PA, which hinders our ability to make definitive comparisons and draw conclusions about the independent risks associated with PA in TAVR, such as postoperative stroke. It remains difficult to ascertain whether the presence of PA itself plays a role for postoperative complications such as stroke or bleeding, or whether these outcomes are instead influenced by other factors related to patient characteristics or procedural techniques. The single-center design also limits the generalizability of our findings, as clinical practices and patient populations may vary across different settings. Furthermore, the small cohort size challenges our ability to detect subtle differences in outcomes.

## 6. Conclusions

Our CT-based analysis has demonstrated that only a minority of patients subjected to TAVR for PA present with full circular calcification of vessel walls. The most extensive calcifications were localized to the outer curvature of aortic arch and the superior aspect of ascending aorta (at origin of brachiocephalic trunk). Patients with circular calcification patterns and those with lesser degrees of alteration displayed similar VARC-3 device success rates, with low short-term mortality and relatively few stroke events. In view of these findings, TAVR constitutes a valid treatment option for patients with PA and AS.

## Figures and Tables

**Table 1:** Patient characteristics and clinical data at baseline.

	Overall (n = 161)	Circular Group (n = 13)	Noncircular Group (n = 148)	*p*-Value
**Patient characteristic:**				
Male sex	107 (66.5%)	7 (53.8%)	100 (67.6%)	0.363
Age, yr	77.2 (70.1–82.6)	76.1 (66.0–86.7)	77.3 (70.3–82.0)	0.818
BMI, kg/m^2^	26.4 (24.4–30.8)	24.8 (22.5–28.2)	26.5 (24.4–30.9)	0.088
Euroscore II	3.10 (1.80–5.58)	2.52 (2.16–4.28)	3.20 (1.75–5.72)	0.748
log Euroscore	11.6 (6.50–21.4)	11.5 (7.6–25.9)	11.6 (6.50–21.4)	0.741
STS predicted mortality risk	2.70 (1.70–4.30)	4.00 (2.00–4.29)	2.70 (1.68–4.35)	0.504
**Clinical parameter:**				
Coronary artery disease, n (%)	54 (33.5%)	6 (46.2%)	48 (32.4%)	0.363
PAD, n (%)	36 (22.4%)	2 (15.4%)	34 (23.0%)	0.734
CAD, n (%)	29 (18.0%)	4 (30.8%)	25 (16.9%)	0.254
Prior PCI, n (%)	53 (32.9%)	5 (38.5%)	48 (32.4%)	0.760
Prior CABG, n (%)	23 (14.3%)	1 (7.69%)	22 (14.9%)	1.000
COPD, n (%)	21 (13.0%)	1 (7.69%)	20 (13.5%)	1.000
History of stroke, n(%)	27 (16.8%)	4 (30.8%)	23 (15.5%)	0.235
Pacemaker, n (%) implant	16 (9.94%)	2 (15.4%)	14 (9.46%)	0.621
Past Radiation, n (%)	19 (11.8%)	5 (38.5%)	14 (9.46%)	0.012
AF, n (%)	49 (30.4%)	4 (30.8%)	45 (30.4%)	1.000
Serum creatinine, mg/dL	1.10 (0.90–1.40)	1.10 (0.80–1.30)	1.10 (0.90–1.40)	0.342

**Table 2:** Calcification scores in aortic segments of patients with porcelain aortas (n = 161).

Score (0–4)	Proximal Segment	Middle Segment	Distal Segment
0	14 (8.7%)	19 (11.8%)	5 (3.1%)
1	51 (31.7%)	40 (24.8%)	15 (9.3%)
2	39 (24.2%)	37 (23%)	34 (21.1%)
3	24 (14.9%)	36 (22.4%)	32 (19.9%)
4	33 (20.5%)	29 (18%)	75 (46.6%)

**Table 3:** Quadrant-level calcification scores of patients with porcelain aortas (n = 161).

Quadrant, %	Score = 0	Score = 1	Score = 2	Score = 3	Score = 4
**Proximal segment**					
Anterior	31.06	11.18	11.18	7.45	9.13
Left	26.71	23.6	14.91	8.07	26.71
Posterior	24.84	21.12	16.15	12.42	25.47
Right	36.65	17.39	6.21	6.21	33.54
**Middle segment**					
Superior	13.04	18.63	18.01	10.56	39.75
Left	37.27	19.88	12.42	8.7	21.74
Posterior	32.92	16.15	13.04	8.07	29.81
Right	25.47	17.39	16.15	8.7	32.3
**Distal segment**					
Superior	4.97	15.53	11.8	12.42	59.01
Left	5.59	8.07	6.83	6.21	73.29
Inferior	11.18	8.7	10.56	10.56	55.28
Right	21.12	18.01	15.53	13.66	31.68

**Table 4:** Procedural data of patients with PA undergoing TAVR procedures.

Parameter	Overall (n = 161)	Circular Group (n = 13)	Noncircular Group (n = 148)	*p*-Value
Intervention time, min	68.0 (50.0–95.0)	58.0 (49.0–70.0)	69.0 (50.0–100)	0.194
Intubation n (%)	86 (53.4%)	4 (30.8%)	82 (55.4%)	0.156
Embolic protection device n (%)	8 (4.97%)	0 (0.00)	8 (5.41%)	1.000
Balloon pre-dilatation, n (%)	49 (30.4%)	1 (7.69%)	48 (32.4%)	0.111
Balloon post-dilatation, n (%)	56 (34.8%)	7 (53.8%)	49 (33.1%)	0.142
Valve position n (%)				1.000
Native AoV	156 (96.9%)	13 (100%)	143 (96.6%)	
AoV bioprosthesis	4 (2.48%)	0 (0.00%)	4 (2.70%)	
Post-AoV-plasty	1 (0.62%)	0 (0.00%)	1 (0.68%)	
Intraoperative pericardial tamponade, n (%)	1 (0.62%)	0 (0.00%)	1 (0.68%)	1.000
Transcatheter heart valve, n (%) *				0.032
BEV	101 (62.7%)	12 (92.3%)	89 (60.1%)	
SEV	60 (37.3%)	1 (7.69%)	59 (39.9%)	
Access route, n (%)				0.938
Transfemoral	121 (75.2%)	10 (76.9%)	111 (75.0%)	
Transapical	22 (13.7%)	2 (15.4%)	20 (13.5%)	
Subclavian	8 (4.97%)	0 (0.00%)	8 (5.41%)	
Transaortal	10 (6.21%)	1 (7.69%)	9 (6.08%)	

* See Supplementary Table S3 for devices used. Periprocedural complications according to VARC-3 criteria were comparable between both patient groups. Overt CNS injury was observed in five patients (3.38%; *p* = 1.0). All patients with perioperative stroke showed a noncircular calcification pattern.

**Table 5:** Periprocedural clinical ramifications of TAVR procedures in patients with PA.

Outcomes	Overall (n = 161)	Circular Group (n = 13)	Noncircular Group (n = 148)	*p*-Value
Device success at discharge, n (%)	138 (85.7%)	13 (100%)	125 (84.5%)	0.218
Postoperative pacemaker implan, n (%)	20 (12.4%)	2 (15.4%)	18 (12.2%)	0.666
Ischemic stroke, n (%)	5 (3.11%)	0 (0.00%)	5 (3.38%)	1.000
Stroke manifestations, n (%)				1.000
<24 h	3 (1.86%)	0 (0.00%)	3 (2.03%)	
24 h–1 month	2 (1.24%)	0 (0.00%)	2 (1.35%)	
Major access complications, n (%)	23 (14.3%)	1 (7.69%)	22 (14.9%)	0.695
Vascular complications, n (%)				0.764
Femoral	22 (13.7%)	1 (7.69%)	21 (14.2%)	
Descendens	1 (0.62%)	0 (0.00%)	1 (0.68%)	
Iliac	1 (0.62%)	0 (0.00%)	1 (0.68%)	
Transapical	1 (0.62%)	0 (0.00%)	1 (0.68%)	
Major bleeding, n (%)	14 (8.70%)	0 (0.00%)	14 (9.46%)	0.607
30-day mortality, n (%)	5 (3.11%)	1 (7.69%)	4 (2.7%)	0.347

**Table 6:** Survival analysis after TAVR in PA patients presenting with circular calcification patterns and noncircular calcification patterns.

Time Interval	Patient Totals	Rate Estimate *	95% CI
**Circular group**			
30 days	12	92.3 ± 7.4%	(78.9–100.0%)
60 days	9	69.2 ± 12.8%	(48.2–99.5%)
90 days	9	69.2 ± 12.8%	(48.2–99.5%)
1 year	9	69.2 ± 12.8%	(48.2–99.5%)
2 years	6	69.2 ± 12.8%	(48.2–99.5%)
**Noncircular group**			
30 days	144	97.3 ± 1.3%	(94.7–99.9%)
60 days	139	93.9 ± 2.0%	(90.1–97.8%)
90 days	135	91.2 ± 2.3%	(86.8–95.9%)
1 year	125	85.8 ± 2.9%	(80.4–91.6%)
2 years	99	80.0 ± 3.3%	(73.7–86.8%)

* percentage ± standard deviation CI, confidence interval.

## Data Availability

The data presented in this study are available on request from the corresponding author due to privacy reasons.

## Supplementary Materials

The following supporting information can be downloaded at: https://www.mdpi.com/article/10.3390/jcm14020503/s1, The supplementary material provides additional information about the pre- and postoperative echocardiographic data of PA patients treated with TAVR. Further, it displays details about the types of aortic valve prostheses used for the TAVR procedure. Table S1: Preoperative echocardiographic data. Table S2: Postoperative echocardiographic data. Table S3: Type of aortic valve prosthesis.

## Abbreviations

AS	aortic stenosis
AF	atrial fibrillation
BEV	balloon-expandable valve
BT	brachiocephalic trunk
CABG	coronary artery bypass graft
CAD	carotid artery disease
CNS	central nervous system
COPD	chronic obstructive lung disease
CPS	cerebral protection system
CT	computed tomography
ICC	intraclass correlation coefficient
MSCT	multi-slice computed tomography
PA	porcelain aorta
PAD	peripheral artery disease
PCI	percutaneous coronary intervention
SEV	self-expandable valve
SAVR	surgical aortic valve replacement
VARC 3	Valve Academic Research Consortium III
TAVR	transcatheter aortic valve replacement
THV	transcatheter heart valve
