# Supplementary material for: Analysis of Calcium Patterns in the Thoracic Aorta and Clinical Outcomes of TAVR Patients Presenting with Porcelain Aorta
Source: J Clin Med. 2025 Jan 14;14(2):503. doi: 10.3390/jcm14020503 (PMC11766034; doi:10.3390/jcm14020503)
Supplement: Supplementary file 1 [file jcm-14-00503-s001.zip › Supplementary Tables.pdf]

Supplement

Table S1. Preoperative echocardiographic Data.

	All	circular	others	P value
**Preop - Echocardiographic data**				
Left ventricular ejection fraction (%)	55.0 [45.0;60.0]	50.0 [48.0;60.0]	56.0 [45.0;60.0]	0.581
Mitral valve stenosis:				0.779
none/minimal	144 (89.4%)	12 (92.3%)	132 (89.2%)	
mild	7 (4.35%)	1 (7.69%)	6 (4.05%)	
moderate	4 (2.48%)	0 (0.00%)	4 (2.70%)	
severe	2 (1.24%)	0 (0.00%)	2 (1.35%)	
Aortic valve mean Pressure Gradient (mmHg)	36.0 [26.0;44.0]	30.0 [26.0;39.0]	36.0 [26.0;44.8]	0.339
Aortic valve maximum Pressure Gradient (mmHg)	57.0 [45.5;72.0]	53.0 [48.8;67.8]	57.0 [45.0;72.5]	0.713
Aortic valve effective Orifice Area (cm2)	0.80 [0.62;0.91]	0.80 [0.69;0.90]	0.80 [0.62;0.92]	0.911
Aortic valve regurgitation:				0.940
none/minimal	66 (41.0%)	5 (38.5%)	61 (41.2%)	
mild	61 (37.9%)	7 (53.8%)	54 (36.5%)	
mild to moderate	9 (5.59%)	0 (0.00%)	9 (6.08%)	
moderate	15 (9.32%)	1 (7.69%)	14 (9.46%)	
moderate to severe	7 (4.35%)	0 (0.00%)	7 (4.73%)	
severe	3 (1.86%)	0 (0.00%)	3 (2.03%)	

Table 2. Post operative echocardiographic data.

	All	circular	others	P value
**Post op - Echocardiographic data**				
Left ventricular ejection fraction (%)	55.0 [48.0;60.0]	55.0 [46.0;62.0]	55.0 [48.0;60.0]	0.808
Mitral valve stenosis:				0.408
none/minimal	129 (80.1%)	9 (69.2%)	120 (81.1%)	
mild	19 (11.8%)	3 (23.1%)	16 (10.8%)	
moderate	3 (1.86%)	0 (0.00%)	3 (2.03%)	
Aortic valve mean Pressure Gradient (mmHg)	9.00 [7.00;12.0]	7.00 [6.00;8.00]	9.00 [7.00;12.0]	0.040
Aortic valve maximum Pressure Gradient (mmHg)	17.0 [13.0;22.0]	14.0 [12.0;17.0]	18.0 [13.0;22.0]	0.060
Aortic valve effective Orifice Area (cm2)	1.70 [1.43;2.00]	1.72 [1.30;1.80]	1.70 [1.44;2.00]	0.573
Aortic valve regurgitation:				1.000
none/minimal	120 (74.5%)	11 (84.6%)	109 (73.6%)	
mild	31 (19.3%)	2 (15.4%)	29 (19.6%)	
mild to moderate	5 (3.11%)	0 (0.00%)	5 (3.38%)	

Table S3. Type of aortic valve prothesis.

	All	circular	others	P value
**Prothesis used n (%):**				0.442
➢ Sapien (23,26,29)	101 (62,73%)	12 (92,31%)	89 (60,14%)	
➢ Evolut (23,26,29,34)	50 (31,06 %)	1 (7,69%)	49 (33,10%)	
➢ Acurate (S,M,L)	5 (3,10 %)	0 (0.00%)	5(3,38%)	
➢ Lotus (23,25,27)	4 (2,48 %)	0 (0.00%)	4 (1.35%)	
➢ Portico 27	1 (0.62%)	0 (0.00%)	1 (0.68%)	
